# Prognostic Stratification of Initial Treatments for Hepatocellular Carcinoma Using a Modified Borderline Resectable Classification

**DOI:** 10.1002/cam4.71470

**Published:** 2025-12-17

**Authors:** Fujimasa Tada, Atsushi Hiraoka, Hideko Ohama, Mai Saito, Yuka Kimura, Ayaka Nakamura, Toru Usui, Kanako Kato, Kei Onishi, Shogo Kitahata, Kozue Kanemitsu‐Okada, Tomoe Kawamura, Taira Kuroda, Naho Ishimura, Jun Hanaoka, Jota Watanabe, Hiromi Ohtani, Teruki Miyake, Osamu Yoshida, Masashi Hirooka, Hideki Miyata, Eiji Tsubouchi, Masanori Abe, Tomoyuki Ninomiya, Yoichi Hiasa

**Affiliations:** ^1^ Gastroenterology Center, Ehime Prefectural Central Hospital Matsuyama Japan; ^2^ Department of Surgery Gastroenterology Center, Ehime Prefectural Central Hospital Matsuyama Japan; ^3^ Department of Gastroenterology and Metabology Ehime University Graduate School of Medicine Toon Japan

**Keywords:** borderline resectable, extrahepatic metastasis, hepatocellular carcinoma, resectability classification

## Abstract

**Aim:**

Although Japanese expert consensus introduced the borderline resectable (BR) criteria, additional classification is considered necessary for clinical practice. This study aimed to evaluate the prognostic predictive value of the modified BR (mBR) criteria.

**Methods:**

From 2009 to 2024, 1056 treatment‐naïve patients with hepatocellular carcinoma (HCC) and Child‐Pugh A were enrolled (median age: 73 years). To obtain the mBR criteria, the original BR classification was modified by separately considering patients with extrahepatic metastasis (EHM, termed “boldly BR”) (*n* = 42) while categorizing the rest into resectable (*n* = 790), mBR1 (*n* = 95), and mBR2 (*n* = 129) groups. Treatments were classified as Cur (curative: surgical resection or radiofrequency ablation), NC (non‐curative: other treatment), or BSC (best supportive care). Clinical features and prognosis according to mBR criteria were analyzed retrospectively.

**Results:**

Overall survival (OS) was stratified according to the mBR criteria (resectable/mBR1/mBR2/BBR = 113.3/49.4/30.1/10.3 months). Albumin‐bilirubin scores worsened progressively, according to the criteria (−2.70/−2.54/−2.40/−2.37), and elevated tumor marker levels (alpha‐fetoprotein [AFP] levels ≥ 100 ng/mL: 18.5%/35.8%/46.5%/59.5%; AFP‐L3 ≥ 10%: 19.8%/40.9%/54.7%/66.7%; and des‐gamma‐carboxy prothrombin levels ≥ 100 mAU/mL: 35.8%/67.4%/81.4%/90.5%, each *p* < 0.001) occurred more frequently in advanced groups. The Cur group in the resectable category showed significantly better OS compared with the NC and BSC groups (121.3/48.1/35.3 months, *p* < 0.001). Similar trends occurred in mBR1 (66.5/34.2/5.2 months) and mBR2 (57.3/22.4/7.5 months). Conversely, although some patients in the curative group had long‐term survival, treatment modality and baseline characteristics showed no prognostic impact on BBR (15.0/10.3/9.0 months).

**Conclusion:**

Other than for patients with EHM, aggressive surgical resection may be considered a potential initial therapeutic option for advanced HCC; however, direct comparisons with other treatment modalities were not performed in this study.

**Abbreviations**
AFP: alpha‐fetoprotein
AFP‐L3:
*lens culinaris* agglutinin‐reactive AFP
ALBI: albumin‐bilirubin
AMED: Japan Agency for Medical Research and Development
BBR: boldly borderline resectable
BCLC: Barcelona Clinic Liver Cancer
BR: borderline resectable
BSC: best supportive care
CI: confidence interval
CT: computed tomography
Cur: curative treatment
DCP: des‐gamma‐carboxy prothrombin
ECOG: Eastern Cooperative Oncology Group
EGV: esophagogastric varices
EHM: extrahepatic metastasis
EZR: Easy R (graphical user interface for R)
HBV: hepatitis B virus
HCC: hepatocellular carcinoma
HCV: hepatitis C virus
HR: hazard ratio
IQR: interquartile range
JSH: Japanese Society of Hepatology
mALBI: modified albumin‐bilirubin
mBR: modified borderline resectable
MRI: magnetic resonance imaging
NA: not applicable
NC: non‐curative treatment
OS: overall survival
PH: portal hypertension
PS: performance status
PVTT: portal vein tumor thrombosis
R: resectable
REGS: recommendation for EGV screening
RFA: radiofrequency ablation
RFS: recurrence‐free survival
SR: surgical resection

## 1 Introduction

Hepatocellular carcinoma (HCC) is the sixth most common cancer and the third or fourth leading cause of cancer‐associated death worldwide [1, 2, 3]. Recent introduction of new systemic therapies has dramatically changed the landscape of the clinical management of advanced HCC. This field is currently receiving considerable attention, with several reports highlighting the therapeutic efficacy of combination therapies or even curative‐intent conversion surgery for patients with advanced HCC [4, 5, 6, 7, 8, 9, 10]. Additionally, unlike surgical resection (SR) for localized intrahepatic tumors, that for extrahepatic metastasis (EHM) is generally considered beneficial only in highly selected patients, in whom intrahepatic tumor is well controlled [11, 12] and/or having oligometastasis with a small number of lesions [13]. However, regarding advanced HCC, the lack of a consensus on the resectability criteria has precluded a constructive discussion on aggressive treatments, including conversion surgery after systemic treatments. Barcelona Clinic Liver Cancer (BCLC) stage recommends non‐curative therapy, such as systemic therapy, for HCC beyond Milan criteria, with portal vein tumor thrombosis (PVTT) positive and/or EHM positive. However, the Japanese Society of Hepatology (JSH) treatment algorithm for HCC recommends surgical resection even for large tumor size and number and/or PVTT. Recently, the Japan Liver Cancer Association and Japanese Society of Hepato‐Biliary‐Pancreatic Surgery launched a working group to discuss new criteria for oncological resectability of HCC. Therefore, a novel definition of oncological resectability of HCC was proposed in 2023 as the borderline resectable (BR) criteria [14]. Although surgical intervention in patients with EHM is infrequently performed, based on the BR criteria, patients are categorized into resectable (R), BR1, and BR2 groups, based on tumor size, number, vascular invasion, and the presence of EHM. However, the benefits of aggressive treatment as an initial therapy in such advanced HCC remain poorly defined.

Accordingly, a modified BR (mBR) classification is necessary. This modification should be based on accumulating evidence including a strong association of intrahepatic control achievement with prolonged survival in patients with HCC [15, 16]. By distinguishing between intrahepatic and extrahepatic factors, the mBR criteria may be able to provide a more rational and clinically meaningful framework for treatment stratification and prognostic assessment, improving upon the somewhat arbitrary boundaries of the original classification.

## 2 Methods

### 2.1 Study Cohort and Inclusion Criteria

**FIGURE 1:**
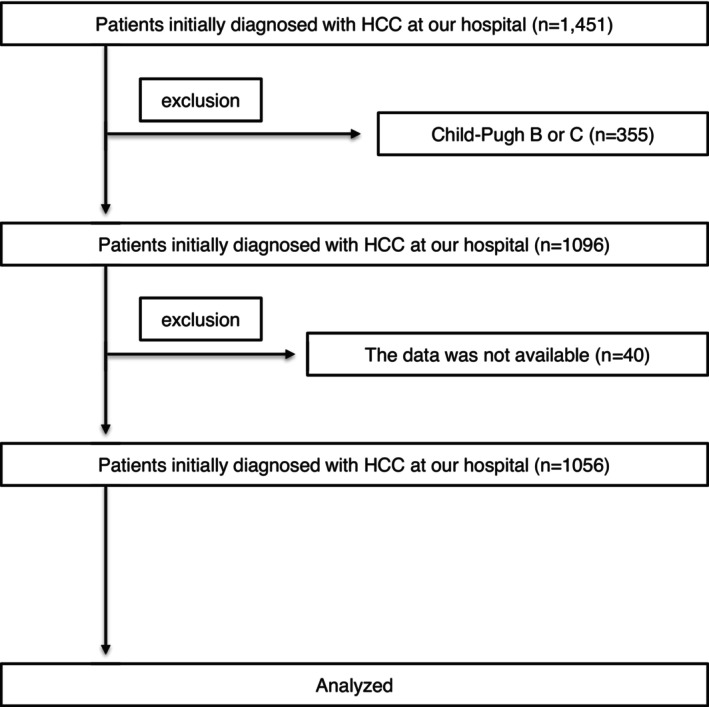
Patient flow diagram of the study cohort. A total of 1451 patients were initially diagnosed with hepatocellular carcinoma at our hospital. After excluding 355 patients with Child‐Pugh class B or C and 40 patients with missing data, 1056 patients were included in the final analysis.

From 2009 to 2024, after excluding patients with missing data or decompensated liver cirrhosis, 1056 patients with HCC, who were initially diagnosed at our institution, were enrolled. At diagnosis, all patients were classified as Child‐Pugh class A and had no prior history of HCC treatment (Figure 1).

### 2.2 mBR Criteria

Patients were categorized according to the mBR criteria, which stratify cases into four groups based on intrahepatic and extrahepatic factors: R, mBR1, mBR2, and boldly borderline resectable (BBR). Any case with EHM was classified as BBR regardless of tumor size, tumor number, or vascular invasion. Among patients without EHM, intrahepatic factors—tumor size, tumor number, and vascular invasion—were used to differentiate R, mBR1, and mBR2, according to the oncological resectability classification proposed by the BR‐HCC Expert Consensus 2023 [14]. Based on these definitions, the 1056 eligible patients were distributed as follows: R (*n* = 790), mBR1 (*n* = 95), mBR2 (*n* = 129), and BBR (*n* = 42) (Table S1).

Patients were further classified by initial treatment strategy: those who underwent SR or radiofrequency ablation (RFA) were included in the curative treatment group (Cur group), while those receiving other treatment modalities were classified into the non‐curative treatment group (NC group). Patients who received no active treatment were categorized into the best supportive care (BSC) group. Clinical background characteristics before treatment and survival outcomes were retrospectively analyzed across these four mBR categories and by treatment group in sub‐analyses.

After obtaining official approval from the Institutional Ethics Committee (no. 26‐11), this study was conducted as a retrospective analysis of database records based on the Guidelines for Clinical Research issued by the Ministry of Health, Labor and Welfare of Japan. All procedures were performed in accordance with the principles of the Declaration of Helsinki. The study adhered to the institutional ethical guidelines, and the requirement for written informed consent was waived due to the retrospective nature of the research, use of anonymized data, and minimal risk to participants. Details of the ethical approval are available on the hospital's official website.

### 2.3 Definition of Portal Hypertension (PH)

To assess PH, an indicator of the significant risk of EGV development, esophagogastric varices (EGV) screening (REGS) score [17], which has been previously validated, was utilized. To evaluate PH prognostic impact, it was defined as a REGS score ≥ 3, which was included in the Cox proportional hazards analysis.

### 2.4 Definition of HCC Etiologies Based on Viral and Alcoholic Markers

Positive anti‐hepatitis C virus (HCV) antibody findings were indicative of HCV‐associated HCC, whereas positive hepatitis B virus (HBV) surface antigen findings were indicative of HBV‐associated HCC. For patients with a history of alcohol abuse (≥ 60 g/day) [18, 19], underlying liver disease was attributed to alcohol consumption.

### 2.5 Assessment of Liver Function and Liver Fibrosis

To assess hepatic reserve function, the Child–Pugh classification [20] and albumin–bilirubin (ALBI) score [21, 22] were used, along with the modified ALBI (mALBI) grade. mALBI grade 2 is further divided into two sub‐grades (2a and 2b) based on the ALBI score cutoff of −2.27 [23].

### 2.6 HCC Diagnosis and Treatment

HCC was diagnosed based on an increase in alpha‐fetoprotein (AFP) level, as well as a dynamic computed tomography (CT) [24], magnetic resonance imaging (MRI) [25, 26], and/or pathological findings obtained during the clinical course.

Since 2005, at our facility, the selected surgical treatment for HCC has primarily been based on the “Guidelines for the Management of Hepatocellular Carcinoma” treatment algorithms, published by the JSH [27, 28, 29, 30, 31]. When SR or ablation was considered as the treatment option, discussions were held during a multidisciplinary team meeting on cases characterized by elevated tumor markers, morphologically aggressive tumor features, or tumors located in anatomically unfavorable sites for ablation. Based on previously published evidence [32], SR was selected as the preferred treatment modality for these cases. In some patients with EHM, SR was actively pursued when the attending physician deemed cancer‐free status achievable, and the patient consented after receiving adequate information.

SR was performed using an open or laparoscopic method.

### 2.7 Follow‐Up After Curative Treatments

Following the curative treatment, imaging (ultrasonography, dynamic CT, or MRI) and tumor marker examinations were performed every 3 to 4 months. When the tumor marker levels increased without a corresponding tumor identification on imaging examination, an alternative imaging modality was used. The time to first recurrence and survival period were recorded and analyzed.

### 2.8 Statistical Analysis

Continuous variables were compared between two groups using the Student's *t*‐test or Welch's *t*‐test, depending on the assumption of equal variances. For non‐normally distributed continuous variables, the Mann–Whitney *U* test was applied. Categorical variables were analyzed using the chi‐square test.

OS and recurrence‐free survival (RFS) were estimated using the Kaplan–Meier method and compared using the log‐rank test. For comparisons among multiple groups, log‐rank *p*‐values were adjusted using the Holm method, to control the family‐wise error rate.

Univariate and multivariate Cox proportional hazards analyses were performed to identify the prognostic factors associated with OS. The following variables (age, sex, etiology [viral vs. non‐viral], mALBI grade, Eastern Cooperative Oncology Group [ECOG] performance status [PS], AFP, *lens culinaris* agglutinin‐reactive AFP [AFP‐L3], des‐gamma‐carboxy prothrombin [DCP], PH [REGS score ≥ 3], and the mBR classification), were included in the univariate analysis based on their known clinical relevance. In addition, the initial treatment strategy was incorporated as a variable, with Cur as the reference category and NC and BSC included for comparison. To explore the potential effect modification, interaction terms between mBR classification and treatment strategy were also included in the multivariate analysis. For the multivariate analysis, all variables were entered simultaneously into the model using a forced‐entry approach, rather than the selection of variables based on the univariate significance.

For non‐normally distributed data, results are presented as medians with interquartile ranges (IQRs). All statistical tests were two‐sided, and a *p*‐value of < 0.05 was considered statistically significant.

All statistical analyses were performed using EZR (version 1.61; Saitama Medical Center, Jichi Medical University, Saitama, Japan) [33], which is a graphical user interface for R (The R Foundation for Statistical Computing, Vienna, Austria).

## 3 Results

The baseline characteristics of this cohort are shown in Table 1. The median age was 73 years (interquartile range: 66–79 years), and 764 patients (72.4%) were male. The ALBI score, a marker of liver reserve function, showed a median of −2.62 (−2.93 to −2.33), and the mALBI grade distributions were as follows: grade 1 in 551 patients (52.2%), grade 2a in 286 (27.1%), grade 2b in 219 (20.7%), and no patients had grade 3.

**TABLE 1:** Overview of baseline characteristics in all enrolled patients (*n* = 1056).

	All (*n* = 1056)
Age, years^a^	73 (66–79)
Sex, male: female	764: 292
Etiology, HCV: HBV: HBV + HCV: Alcohol: NBNC	528: 103: 9: 142: 274
R: mBR1: mBR2: BBR	790: 95: 129: 42
BMI, kg/m^2a^	23.3 (21.3–25.6)
ECOG PS, 0: 1: 2: 3: 4	816: 141: 49: 36: 14
ALBI score^a^	−2.62 (−2.93 to −2.33)
mALBI grade 1:2a:2b:3	551: 286: 219: 0
AST, U/L^a^	41 (28–64)
ALT, U/L^a^	33 (21–54)
Platelets, 10^4^/μL^a^	14.2 (10.2–18.8)
Total bilirubin, mg/dL^a^	0.7 (0.6–1.0)
Albumin, g/dL^a^	4.0 (3.6–4.3)
Prothrombin time, %^a^	89.0 (80.0–99.0)
Tumor size (maximum), cm^a^	2.8 (1.7–4.7)
Tumor diameter ≥ 2 cm, *n* (%)	720 (68.2)
Tumor number, single: multiple	703: 353
Portal invasion (vp0/vp1/vp2/vp3/vp4), *n* (%)	984/11/24/20/17 (93.2/1.0/2.3/1.9/1.6)
Venous invasion (vv0/vv1/vv2/vv3), *n* (%)	1039/2/8/7/0 (98.4/0.2/0.8/0.7)
Biliary invasion (b0/b1/b2/b3/b4), *n* (%)	1053/1/1/1/0 (99.7/0.1/0.1/0.1/0)
AFP, ng/mL^a^	10.5 (4.4–103.0)
AFP ≥ 100 ng/mL, *n* (%)	265 (25.1)
AFP‐L3, %^a^	3.5 (0.5–14.8)
AFP‐L3 ≥ 10%, *n* (%)	287 (28.0)
DCP, mAU/mL^a^	78.0 (27.0–947.5)
DCP ≥ 100 mAU/mL, *n* (%)	490 (46.4)
Presence of EHM, *n* (%)	42 (4.0)
EHM in original BR (BR1/BR2), *n* (%)	3/39 (7.1/92.9)
Sites of EHM (bone/lymph node/lung/mediastinum/pulmonary artery/peritoneum/lt adrenal gland), *n* (%)	16/14/8/1/1/1/1 (38.1/33.3/19.0/2.3/2.3/2.3/2.3)
Cur: non‐Cur: BSC	812: 151: 93
Surgical resection: RFA	360: 452

Abbreviations: AFP, alpha‐fetoprotein; AFP‐L3, *lens culinaris* agglutinin‐reactive AFP; ALBI score, albumin‐bilirubin score; ALT, alanine aminotransferase; AST, aspartate aminotransferase; BBR, boldly borderline resectable; BMI, body mass index; BR, borderline resectable; BSC, best supportive care; Cur, curative therapy; DCP, des‐gamma‐carboxy prothrombin; ECOG PS, Eastern Cooperative Oncology Group performance status; EHM, extrahepatic metastasis; HBV, hepatitis B virus; HCV, hepatitis C virus; mALBI grade, modified ALBI grade; mBR, modified borderline resectable; NBNC, non‐HBV‐non‐HCV; R, resectable; RFA, radiofrequency ablation.

^a^ Median (interquartile range).

The median maximum tumor diameter was 2.8 cm (1.7–4.7), and 68.2% of patients developed tumors ≥ 2 cm. Single and multiple tumors were observed in 703 and 353 patients, respectively. Elevated AFP (≥ 100 ng/mL), AFP‐L3 (≥ 10%), and DCP (≥ 100 mAU/mL) were observed in 25.1%, 28.0% and 46.4% of patients, respectively. Portal, venous, and biliary invasions were observed in 82 (7.8%), 17 (1.6%), and 3 (0.3%) patients, respectively. EHM was identified in 42 patients (4.0%). The most common sites of EHM were the bone (16 cases, 38.1%); lymph nodes (14 cases, 33.3%); and lungs (8 cases, 19.0%). Less frequent sites included the mediastinum, pulmonary artery, peritoneum, and left adrenal gland, observed in one case each. According to the original BR classification, 3 (7.1%) and 39 (92.9%) patients were categorized into BR1 and BR2 groups, respectively. Among the BR1 group, EHM included one case each of pulmonary metastases (≤ 3 nodules), left adrenal metastasis, and a solitary hilar lymph node metastasis.

Of 812 patients who underwent curative treatments, 360 underwent SR and 452 underwent RFA. However, non‐curative treatments were administered to 151 patients, while 93 patients received BSC.

**FIGURE 2:**
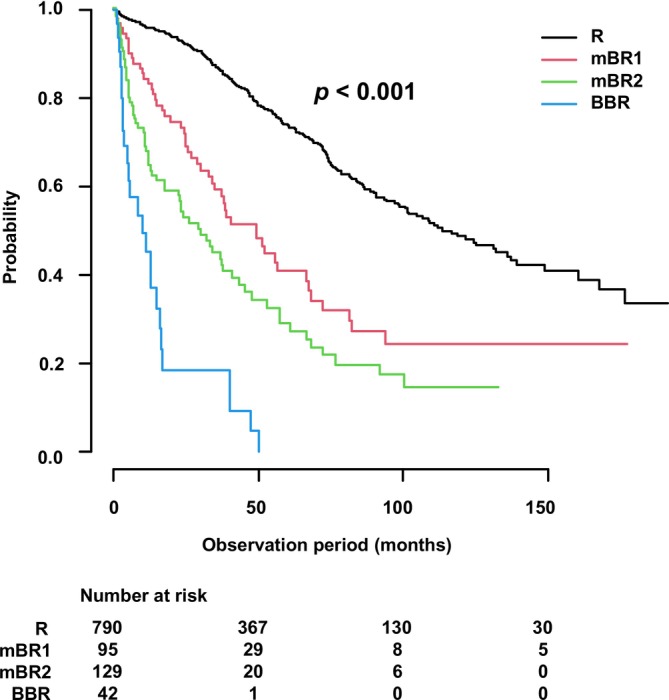
Survival outcomes according to modified borderline resectable (mBR) criteria. Overall survival (OS) progressively worsened with advancing mBR classification (median OS: R vs. mBR1 vs. mBR2 vs. BBR = 113.3 vs. 49.4 vs. 30.1 vs. 10.3 months). A significant difference was observed among the four groups (log‐rank test, *p* < 0.001). Results of pairwise comparisons using the Holm method are shown in Table S2. BBR, boldly borderline resectable; BR, borderline resectable; mBR, modified borderline resectable; OS, overall survival; R, resectable.

OS decreased progressively with advancing mBR classification. The median OS [mOS] (95% confidence interval [CI]) was 113.3 months [100.6–136.0] in the R group; 49.4 months [34.2–66.5] in mBR1; 30.1 months [17.6–41.1] in mBR2; and 10.3 months [3.7–15.9] in BBR (*p* < 0.001) (Figure 2). Subsequent pairwise comparisons adjusted using the Holm method revealed statistically significant differences between all group pairs: R versus mBR1 (*p* < 0.001); R versus mBR2 (*p* < 0.001); R versus BBR (p < 0.001); mBR1 versus mBR2 (*p* = 0.025); mBR1 versus BBR (*p* < 0.001); and mBR2 versus BBR (*p* < 0.001) (Table S2). These findings indicate distinct survival distribution patterns among all groups.

Baseline clinical characteristics for each mBR class are shown in Table 2. In addition to tumor‐related factors, other clinical parameters also significantly differed among the four mBR classes. Serum albumin levels and prothrombin time significantly declined with advancing mBR class (each *p* < 0.001). Consequently, liver function worsened (median ALBI score: R vs. mBR1 vs. mBR2 vs. BBR = −2.70 vs. −2.54 vs. −2.40 vs. −2.37), with deteriorating mALBI grade distribution (each *p* < 0.001).

**TABLE 2:** Baseline clinical characteristics of four patient groups defined by modified borderline resectable (mBR) criteria.

	*R* (*n* = 790)	mBR1 (*n* = 95)	mBR2 (*n* = 129)	BBR (*n* = 42)	*p*
Age, years^a^	73 (66–79)	74 (66–80)	73 (66–79)	75 (64–78)	0.931
Sex, male: female	564: 226	66: 29	99: 30	35: 7	0.209
Etiology, HCV: HBV: HBV + HCV: Alcohol: NBNC	410: 77: 7: 100: 196	52: 11: 1: 12:19	52: 9: 1: 24: 43	14: 6: 0: 6: 16	0.11
BMI, kg/m^2^ ^a^	23.3 (21.2–25.5)	22.9 (21.4–24.9)	23.7 (22.2–26.4)	22.9 (19.9–26.4)	0.096
ECOG PS, 0: 1: 2: 3: 4	631: 94: 28: 28: 9	66: 21: 4:3: 1	94: 15: 14: 5: 1	25: 11: 3: 0: 3	< 0.001
PS 0/1: 2/3/4, (%)	725: 65 (91.8: 8.2)	87: 8 (91.6: 8.4)	109: 20 (84.5: 15.5)	36: 6 (85.7: 14.3)	0.042
ALBI score^a^	−2.70 (−2.99 to −2.36)	−2.54 (−2.85 to −2.35)	−2.40 (−2.65 to −2.14)	−2.37 (−2.69 to −2.17)	< 0.001
mALBI grade 1:2a:2b:3	459: 195: 136: 0	38: 38: 19: 0	40: 41: 48: 0	14: 12: 16: 0	< 0.001
AST, U/L^a^	37 (26–58)	48 (32–76)	60 (41–87)	59 (37–88)	< 0.001
ALT, U/L^a^	30 (19–49)	43 (25–64)	43 (27–64)	38 (27–59)	< 0.001
Platelets, 10^4^/μL^a^	13.9 (9.9–18.2)	13.5 (9.8–17.2)	15.7 (11.2–21.2)	20.6 (14.9–26.9)	< 0.001
Total bilirubin, mg/dL^a^	0.7 (0.5–1.0)	0.7 (0.5–1.2)	0.8 (0.6–1.1)	0.7 (0.6–1.2)	0.017
Albumin, g/dL^a^	4.0 (3.7–4.3)	3.9 (3.6–4.1)	3.7 (3.4–4.0)	3.6 (3.5–4.0)	< 0.001
Prothrombin time, %^a^	90.0 (80.9–100.0)	88.4 (81.3–95.5)	89.0 (80.0–96.5)	82.0 (75.0–88.8)	< 0.001
Tumor size (maximum), cm^a^	2.3 (1.5–3.5)	3.9 (3.2–4.5)	6.2 (4.5–9.0)	7.3 (5.4–11.2)	< 0.001
Tumor diameter ≥ 2 cm, *n* (%)	470 (59.5)	87 (91.6)	121 (93.8)	42 (100)	< 0.001
Tumor number, single: multiple	672: 118	13: 82	7: 122	11: 31	< 0.001
Portal invasion (vp0/vp1/vp2/vp3/vp4), *n* (%)	784/6/0/0/0 (99.2/0.8/0/0/0)	75/2/12/6/0 (78.9/2.1/12.6/6.3/0)	93/1/10/10/15 (72.1/0.8/7.8/7.8/11.6)	32/2/2/4/2 (76.2/4.8/4.8/9.5/4.8)	< 0.001
Venous invasion (vv0/vv1/vv2/vv3), *n* (%)	789/1/0/0 (99.9/0.1/0/0)	91/1/3/0 (95.8/1.1/3.2/0)	123/0/4/2 (95.3/0/3.1/1.6)	36/0/1/5 (85.7/0/2.4/11.9)	< 0.001
Biliary invasion (b0/b1/b2/b3/b4), *n* (%)	790/0/0/0/0 (100/0/0/0/0)	95/0/0/0/0 (100/0/0/0/0)	126/1/1/1/0 (97.7/0.8/0.8/0.8/0)	42/0/0/0/0 (100/0/0/0/0)	0.01
AFP, ng/mL^a^	7.8 (3.8–45.9)	29.0 (6.4–214.1)	69.3 (11.1–2314.0)	308.5 (25.2–4723.7)	< 0.001
AFP ≥ 100 ng/mL, *n* (%)	146 (18.5)	34 (35.8)	60 (46.5)	25 (59.5)	< 0.001
AFP‐L3, %^a^	1.4 (0.5–8.1)	6.4 (0.5–26.8)	16.3 (3.7–53.8)	22.4 (8.9–49.2)	< 0.001
AFP‐L3 ≥ 10%, *n* (%)	151 (19.8)	38 (40.9)	70 (54.7)	28 (66.7)	< 0.001
DCP, mAU/mL^a^	49 (24–237)	430 (74–2537)	2509 (314–12,960)	24,652 (2987–40,564)	< 0.001
DCP ≥ 100 mAU/mL, *n* (%)	283 (35.8)	64 (67.4)	105 (81.4)	38 (90.5)	< 0.001
Cur: non‐Cur: BSC	708: 33: 49	60: 25: 10	37: 68: 24	7: 25: 10	< 0.001
Surgical resection: RFA	356: 352	52: 8	37: 0	7:0	< 0.001

Abbreviations: AFP, alpha‐fetoprotein; AFP‐L3, *lens culinaris* agglutinin‐reactive AFP; ALBI score, albumin‐bilirubin score; ALT, alanine aminotransferase; AST, aspartate aminotransferase; BBR, boldly borderline resectable; BMI, body mass index; BR, borderline resectable; BSC, best supportive care; Cur, curative therapy; DCP, des‐gamma‐carboxy prothrombin; ECOG PS, Eastern Cooperative Oncology Group performance status; EHM, extrahepatic metastasis; HBV, hepatitis B virus; HCV, hepatitis C virus; mALBI grade, modified ALBI grade; mBR, modified borderline resectable; NBNC, non‐HBV‐non‐HCV; R, resectable; RFA, radiofrequency ablation.

^a^ Median (interquartile range).

The proportion of patients with PS ≥ 2 was significantly higher in the mBR2 and BBR groups compared to those in R and mBR1 groups (PS ≥ 2: R vs. mBR1 vs. mBR2 vs. BBR = 8.2% vs. 8.4% vs. 15.5% vs. 14.3%, *p* = 0.042). Furthermore, elevated tumor marker levels (AFP, AFP‐L3, and DCP) increased with advancing mBR classification (each *p* < 0.001).

**FIGURE 3:**
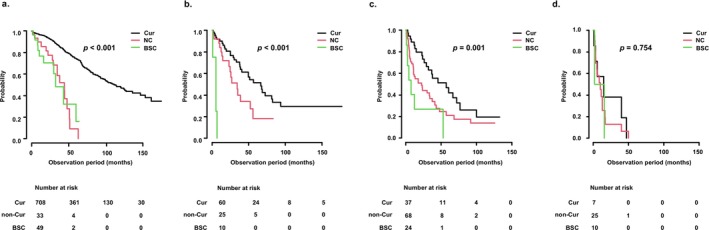
Overall survival according to treatment intensity across modified borderline resectable (mBR) classification subgroups. (a) In the resectable (R) group, overall survival (OS) was significantly longer in the curative treatment (Cur) group compared to the non‐curative (NC) and best supportive care (BSC) groups (median OS: 121.3 vs. 48.1 vs. 35.3 months, respectively; *p* < 0.001). (b) In the mBR1 group, OS was significantly longer in the Cur group compared to the NC and BSC groups (median OS: 66.5 vs. 34.2 vs. 5.2 months, respectively; *p* < 0.001). (c) In the mBR2 group, OS was also significantly longer in the Cur group compared to the NC and BSC groups (median OS: 57.4 vs. 22.4 vs. 7.5 months, respectively; *p* < 0.001). (d) In contrast, in the BBR group, no significant difference in OS was observed among the treatment groups (median OS: 15.0 vs. 10.3 vs. 9.0 months, respectively; *p* = 0.754). BBR, boldly borderline resectable; BSC, best supportive care; Cur, curative treatment; mBR, modified borderline resectable; NC, non‐curative treatment; OS, overall survival; R, resectable.

In a sub‐analysis based on treatment intensity, OS was significantly longer in the Cur group than in the NC and BSC groups among patients in the R group (mOS [95% CI]: Cur vs. NC vs. BSC = 121.3 [104.5–148.7] vs. 48.1 [31.6–55.9] vs. 35.3 [17.6–65.4] months, *p* < 0.001; Figure 3a). Patients in the Cur group were younger (median age: 72 vs. 78 vs. 82 years); had a higher proportion of PS 0 or 1 (94.8% vs. 75.8% vs. 59.2%); and better liver function based on the ALBI score (−2.73 vs. −2.36 vs. −2.42; all *p* < 0.001; Table 3). Similar trends were observed in the mBR1 group (mOS [95% CI]: 66.5 [38.8–82.4] vs. 34.2 [14.6–52.0] vs. 5.2 [0.7–not applicable (NA)] months; Figure 3b) and the mBR2 group (57.4 [29.4–76.7] vs. 22.4 [10.9–34.0] vs. 7.5 [2.3–NA] months; Figure 3c), with each comparison showing *p* < 0.001. Baseline characteristics were also comparable among treatment groups in these strata (Tables S3 and S4). In contrast, there were no significant differences in OS among the treatment groups in the BBR cohort (mOS [95% CI]: 15.0 [1.2–NA] vs. 10.3 [3.4–13.0] vs. 9.0 [2.1–NA] months, *p* = 0.754; Figure 3d), and the baseline characteristics were similarly distributed among the three groups (Table 4).

**TABLE 3:** Baseline clinical characteristics of patients in the resectable group stratified by treatment strategy based on modified borderline resectable (mBR) criteria.

	Curative (*n* = 708)	Non‐curative (*n* = 33)	BSC (*n* = 49)	*p*
Age, years^a^	72 (65–78)	78 (70–81)	82 (78–86)	< 0.001
Sex, male: female	510: 198	21: 12	33: 16	0.471
Etiology, HCV: HBV: HBV + HCV: Alcohol: NBNC	367: 73: 7: 87: 174	17: 1: 0: 5: 10	26: 3: 0: 8: 12	0.817
BMI, kg/m^2^ ^a^	23.2 (21.2–25.4)	23.9 (21.6–27.0)	23.4 (21.9–25.8)	0.437
ECOG PS, 0: 1: 2: 3: 4	592: 79: 18: 17: 2	20: 5: 3:4: 1	19: 10: 7: 7: 6	< 0.001
PS 0/1: 2/3/4, (%)	671: 37 (94.8: 5.2)	25: 8 (75.8: 24.2)	29: 20 (59.2: 40.8)	< 0.001
ALBI score^a^	−2.73 (−2.99 to −2.43)	−2.36 (−2.61 to −2.26)	−2.42 (−2.76 to −2.25)	< 0.001
mALBI grade 1:2a:2b:3	433: 162: 113: 0	9: 15: 9: 0	17: 18: 14: 0	< 0.001
AST, U/L^a^	37 (25–58)	44 (32–57)	36 (28–62)	0.31
ALT, U/L^a^	31 (20–49)	28 (22–46)	26 (18–44)	0.553
Platelets, 10^4^/μL^a^	13.9 (10.1–18.0)	12.7 (8.1–19.3)	16.3 (10.6–21.3)	0.095
Total bilirubin, mg/dL^a^	0.7 (0.5–1.0)	0.9 (0.6–1.0)	0.8 (0.6–1.1)	0.088
Albumin, g/dL^a^	4.1 (3.7–4.3)	3.7 (3.5–3.9)	3.8 (3.5–4.0)	< 0.001
Prothrombin time, %^a^	90.0 (81.0–100.0)	84.0 (76.0–93.5)	89.3 (77.0–101.0)	0.061
Tumor size (maximum), cm^a^	2.2 (1.5–3.4)	2.8 (1.9–5.3)	3.5 (2.2–4.6)	< 0.001
Tumor diameter ≥ 2 cm, *n* (%)	407 (57.5)	24 (72.7)	39 (79.6)	0.003
Tumor number, single: multiple	602: 106	24: 9	46: 3	0.031
Portal invasion (vp0/vp1/vp2/vp3/vp4), *n* (%)	702/6/0/0/0 (99.2/0.8/0/0/0)	33/0/0/0/0 (100/0/0/0/0)	49/0/0/0/0 (100/0/0/0/0)	0.705
Venous invasion (vv0/vv1/vv2/vv3), *n* (%)	708/0/0/0 (100/0/0/0)	33/0/0/0 (100/0/0/0)	48/1/0/0 (98.0/2.0/0/0)	0.001
Biliary invasion (b0/b1/b2/b3/b4), *n* (%)	708/0/0/0/0 (100/0/0/0/0)	33/0/0/0/0 (100/0/0/0/0)	49/0/0/0/0 (100/0/0/0/0)	NA
AFP, ng/mL^a^	7.5 (3.8–40.4)	7.8 (5.6–49.4)	14.7 (3.9–95.6)	0.082
AFP ≥ 100 ng/mL, *n* (%)	128 (18.1)	6 (18.2)	12 (24.5)	0.535
AFP‐L3, %^a^	1.0 (0.5–7.8)	4.1 (0.5–17.3)	5.2 (0.5–20.1)	0.041
AFP‐L3 ≥ 10%, *n* (%)	129 (18.8)	10 (32.3)	12 (27.3)	0.08
DCP, mAU/mL^a^	45 (23–176)	74 (25–2000)	345 (31–1230)	< 0.001
DCP ≥ 100 mAU/mL, *n* (%)	236 (33.3)	15 (45.5)	32 (65.3)	< 0.001

Abbreviations: AFP, alpha‐fetoprotein; AFP‐L3, *lens culinaris* agglutinin‐reactive AFP; ALBI score, albumin‐bilirubin score; ALT, alanine aminotransferase; AST, aspartate aminotransferase; BBR, boldly borderline resectable; BMI, body mass index; BR, borderline resectable; BSC, best supportive care; Cur, curative therapy; DCP, des‐gamma‐carboxy prothrombin; ECOG PS, Eastern Cooperative Oncology Group performance status; EHM, extrahepatic metastasis; HBV, hepatitis B virus; HCV, hepatitis C virus; mALBI grade, modified ALBI grade; NA, not available; NBNC, non‐HBV‐non‐HCV; R, resectable; RFA, radiofrequency ablation.

^a^ Median (interquartile range).

**TABLE 4:** Baseline clinical characteristics of patients in the boldly borderline resectable (BBR) group stratified by treatment strategy based on modified borderline resectable (mBR) criteria.

	Curative (*n* = 7)	Non‐curative (*n* = 25)	BSC (*n* = 10)	*p*
Age, years^a^	72 (62–74)	77 (66–79)	72 (57–77)	0.356
Sex, male: female	7: 0	20: 5	8: 2	0.432
Etiology, HCV: HBV: HBV + HCV: Alcohol: NBNC	5: 1: 0: 1: 0	8: 3: 0: 4: 10	1: 2: 0: 1: 6	0.166
BMI, kg/m^2^ ^a^	23.4 (20.1–26.2)	22.9 (19.7–26.3)	21.9 (19.3–25.3)	0.911
ECOG PS, 0: 1: 2: 3: 4	5: 1: 1 0: 0	16: 8: 0:0: 1	4: 2: 2: 0: 2	0.147
PS 0/1: 2/3/4, (%)	6: 1 (85.7: 14.3)	24: 1 (96.0: 4.0)	6: 4 (60.0: 40.0)	0.023
ALBI score^a^	−2.62 (−2.75 to −2.43)	−2.28 (−2.69 to −2.16)	−2.34 (−2.39 to −2.15)	0.284
mALBI grade 1:2a:2b:3	4: 2: 1: 0	8: 6: 11: 0	2: 4: 4: 0	0.454
AST, U/L^a^	54 (45–62)	61 (37–98)	60 (34–84)	0.797
ALT, U/L^a^	49 (34–60)	36 (28–54)	36 (24–49)	0.794
Platelets, 10^4^/μL^a^	15.3 (11.1–25.5)	20.7 (14.8–24.9)	22.3 (18.6–29.7)	0.505
Total bilirubin, mg/dL^a^	0.7 (0.6–1.1)	0.9 (0.6–1.3)	0.6 (0.6–0.7)	0.198
Albumin, g/dL^a^	4.0 (3.6–4.2)	3.6 (3.5–4.0)	3.5 (3.2–3.6)	0.223
Prothrombin time, %^a^	79.9 (67.9–88.6)	83.0 (75.7–91.0)	79.5 (75.8–86.3)	0.643
Tumor size (maximum), cm^a^	8.0 (6.3–9.9)	6.0 (5.0–11.0)	8.1 (6.8–12.2)	0.512
Tumor diameter ≥ 2 cm, *n* (%)	7 (100)	25 (100)	10 (100)	NA
Tumor number, single: multiple	3: 4	6: 19	2: 8	0.531
Portal invasion (vp0/vp1/vp2/vp3/vp4), *n* (%)	6/0/0/1/0 (85.7/0/0/14.3/0)	21/0/1/1/2 (84.0/0/4.0/4.0/8.0)	5/2/1/2/0 (50.0/20.0/10.0/20.0/0)	0.151
Venous invasion (vv0/vv1/vv2/vv3), *n* (%)	7/0/0/0 (100/0/0/0)	22/0/0/3 (88.0/0/0/12.0)	7/0/1/2/0 (70.0/0/10.0/20.0/0)	0.282
Biliary invasion (b0/b1/b2/b3/b4), *n* (%)	7/0/0/0/0 (100/0/0/0/0)	25/0/0/0/0 (100/0/0/0/0)	10/0/0/0/0 (100/0/0/0/0)	NA
AFP, ng/mL^a^	124.6 (51.5–9650.2)	603.7 (26.5–4883.2)	111.9 (20.2–1540.9)	0.84
AFP ≥ 100 ng/mL, *n* (%)	4 (57.1)	16 (64.0)	5 (50.0)	0.741
AFP‐L3, %^a^	33.5 (15.4–63.8)	26.0 (9.7–52.2)	12.3 (4.4–27.3)	0.276
AFP‐L3 ≥ 10%, *n* (%)	5 (71.4)	17 (68.0)	6 (60.0)	0.864
DCP, mAU/mL^a^	14,388 (7686–33,448)	23,237 (3201–37,669)	29,481 (1041–67,403)	0.918
DCP ≥ 100 mAU/mL, *n* (%)	7 (100.0)	23 (92.0)	8 (80.0)	0.354

Abbreviations: AFP, alpha‐fetoprotein; AFP‐L3, *lens culinaris* agglutinin‐reactive AFP; ALBI score, albumin‐bilirubin score; ALT, alanine aminotransferase; AST, aspartate aminotransferase; BBR, boldly borderline resectable; BMI, body mass index; BR, borderline resectable; BSC, best supportive care; DCP, des‐gamma‐carboxy prothrombin; ECOG PS, Eastern Cooperative Oncology Group performance status; HBV, hepatitis B virus; HCV, hepatitis C virus; mALBI grade, modified ALBI grade; mBR, modified borderline resectable; NA, not available; NBNC, non‐HBV‐non‐HCV; RFA, radiofrequency ablation.

^a^ Median (interquartile range).

Among patients who underwent curative treatments, recurrence was observed in 71.7% (43/60) and 70.3% (26/37) of those classified as having mBR1 and mBR2, respectively, with no significant difference between the groups (*p* = 1.000). For the mBR1 and mBR2 groups, the recurrence rates within 1 year were 44.2% vs. 69.2% and within 2 years were 83.7% vs. 88.5%, respectively. Although the 1‐year recurrence rate tended to be lower in the mBR1 group, this difference did not reach statistical significance (*p* = 0.051). There was also no significant difference in the 2‐year recurrence rates between the two groups (*p* = 0.732).

**FIGURE 4:**
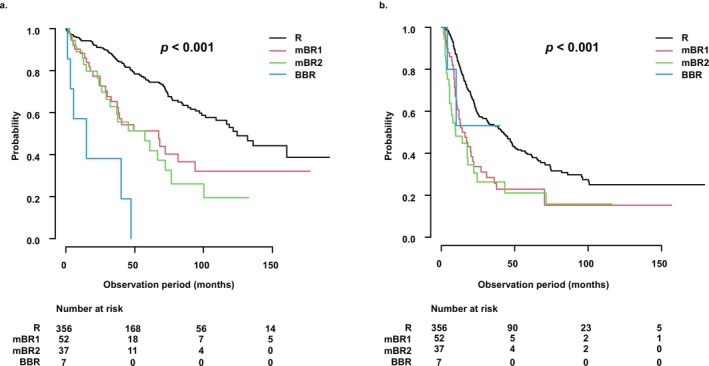
Survival and recurrence outcomes in HCC patients treated with surgical resection according to the modified borderline resectable (mBR) criteria. (a) Among patients who underwent surgical resection (R: *N* = 356; mBR1: *N* = 52; mBR2: *N* = 37; BBR: *N* = 7), overall survival (OS) progressively declined with worsening mBR classification (median OS: R vs. mBR1 vs. mBR2 vs. BBR = 124.2 vs. 67.4 vs. 57.4 vs. 15.0 months; *p* < 0.001). (b) Similarly, recurrence‐free survival (RFS) also significantly decreased across the mBR subgroups (median RFS: R vs. mBR1 vs. mBR2 vs. BBR = 41.9 vs. 14.5 vs. 9.9 vs. not applicable [NA]; *p* < 0.001). BBR, boldly borderline resectable; HCC, hepatocellular carcinoma; mBR, modified borderline resectable; NA, not applicable; OS, overall survival; R, resectable; RFS, recurrence‐free survival; SR, surgical resection.

In a sub‐analysis limited to patients who underwent SR (R: *n* = 356; mBR1: *n* = 52; mBR2: *n* = 37; BBR: *n* = 7), OS progressively worsened with increasing mBR classification severity (mOS [95% CI]: R vs. mBR1 vs. mBR2 vs. BBR = 124.2 [101.3–NA] vs. 67.5 [33.0–93.7] vs. 57.4 [29.4–76.7] vs. 15.0 [1.2‐NA] months, *p* < 0.001) (Figure 4a). RFS showed a similar trend (median RFS [95% CI]: R vs. mBR1 vs. mBR2 vs. BBR = 41.9 [31.4–48.5] vs. 14.5 [10.8–21.7] vs. 9.9 [5.7–22.1] vs. NA [4.3‐NA] months, *p* < 0.001) (Figure 4b). Besides the tumor‐related factors and laboratory values, such as aspartate aminotransferase, alanine aminotransferase, and platelet count, no significant differences were observed in baseline clinical characteristics among the groups (Table 5).

**TABLE 5:** Baseline clinical characteristics of surgical cases stratified by modified borderline resectable (mBR) criteria.

	R (*n* = 356)	mBR1 (*n* = 52)	mBR2 (*n* = 37)	BBR (*n* = 7)	*p*
Age, years^a^	73 (66–78)	71 (64–79)	67 (62–75)	72 (62–74)	0.119
Sex, male: female	284: 72	39: 13	30: 7	7: 0	0.47
Etiology, HCV: HBV: HBV + HCV: Alcohol: NBNC	151: 38: 3: 44: 120	28: 10: 1: 6: 7	14: 1: 0: 8: 14	5: 1: 0: 1: 0	0.059
BMI, kg/m^2^ ^a^	23.6 (21.4–25.6)	22.9 (21.4–24.7)	24.1 (22.1–26.7)	23.4 (20.1–26.2)	0.333
ECOG PS, 0: 1: 2: 3: 4	291: 45: 10: 9: 1	40: 11: 0:1: 0	33: 4: 0: 0: 0	5: 1: 1: 0: 0	0.531
PS 0/1: 2/3/4, (%)	336: 20 (94.4: 5.6)	51: 1 (98.1: 1.9)	37: 0 (100: 0)	6: 1 (85.7: 14.3)	0.2
ALBI score^a^	−2.76 (−3.04 to −2.52)	−2.64 (−2.91 to −2.48)	−2.61 (−2.74 to −2.43)	−2.62 (−2.75 to −2.43)	0.057
mALBI grade 1:2a:2b:3	229: 84: 43: 0	27: 20: 5: 0	21: 10: 6: 0	4: 2: 1: 0	0.411
AST, U/L^a^	33 (24–53)	46 (29–68)	55 (38–78)	54 (45–62)	< 0.001
ALT, U/L^a^	28 (19–47)	44 (21–63)	43 (27–64)	49 (34–60)	0.007
Platelets, 10^4^/μL^a^	15.3 (11.6–19.8)	13.5 (9.7–17.2)	18.9 (14.9–24.2)	15.3 (11.0–25.5)	0.001
Total bilirubin, mg/dL^a^	0.7 (0.5–0.9)	0.7 (0.4–0.9)	0.7 (0.6–0.9)	0.7 (0.6–1.1)	0.452
Albumin, g/dL^a^	4.1 (3.8–4.4)	4.0 (3.8–4.2)	3.9 (3.7–4.1)	4.0 (3.6–4.2)	0.022
Prothrombin time, %^a^	92.3 (83.0–102.0)	89.0 (84.5–94.9)	88.0 (80.0–95.4)	79.9 (67.9–88.6)	0.015
Tumor size (maximum), cm^a^	3.3 (2.2–4.8)	4.0 (3.5–4.6)	8.0 (6.0–10.0)	8.0 (6.3–9.9)	< 0.001
Tumor diameter ≥ 2 cm, *n* (%)	278 (78.1)	52 (100)	36 (97.3)	7 (100)	< 0.001
Tumor number, single: multiple	326: 30	8: 44	1: 36	3: 4	< 0.001
Portal invasion (vp0/vp1/vp2/vp3/vp4), *n* (%)	350/6/0/0/0 (98.3/1.7/0/0/0)	41/1/8/2/0 (78.8/1.9/15.4/3.8/0)	30/1/4/1/1 (81.1/2.7/10.8/2.7/2.7)	6/0/0/1/0 (85.7/0/0/14.3/0)	< 0.001
Venous invasion (vv0/vv1/vv2/vv3), *n* (%)	356/0/0/0 (100/0/0/0)	51/1/0/0 (98.1/1.9/0/0)	36/0/1/0 (97.3/0/2.7/0)	7/0/0/0 (100/0/0/0)	0.004
Biliary invasion (b0/b1/b2/b3/b4), *n* (%)	356/0/0/0/0 (100/0/0/0/0)	52/0/0/0/0 (100/0/0/0/0)	37/0/0/0/0 (100/0/0/0/0)	7/0/0/0/0 (100/0/0/0/0)	NA
AFP, ng/mL^a^	7.4 (3.8–58.8)	31.9 (7.0–214.0)	60.6 (11.1–2140.6)	124.6 (51.5–9650.2)	< 0.001
AFP ≥ 100 ng/mL, *n* (%)	79 (22.2)	18 (34.6)	16 (43.2)	4 (57.1)	0.003
AFP‐L3, %^a^	1.2 (0.5–13.3)	7.4 (1.5–37.0)	25.2 (6.5–54.6)	33.5 (15.4–63.8)	< 0.001
AFP‐L3 ≥ 10%, *n* (%)	83 (24.6)	24 (47.1)	21 (56.8)	5 (71.4)	< 0.001
DCP, mAU/mL^a^	85 (31–649)	721 (126–2729)	7141 (768–22,972)	14,388 (7686–33,448)	< 0.001
DCP ≥ 100 mAU/mL, *n* (%)	167 (46.9)	40 (76.9)	35 (94.6)	7 (100)	< 0.001

Abbreviations: AFP, alpha‐fetoprotein; AFP‐L3, *lens culinaris* agglutinin‐reactive AFP; ALBI score, albumin‐bilirubin score; ALT, alanine aminotransferase; AST, aspartate aminotransferase; BBR, boldly borderline resectable; BMI, body mass index; BR, borderline resectable; BSC, best supportive care; DCP, des‐gamma‐carboxy prothrombin; ECOG PS, Eastern Cooperative Oncology Group performance status; HBV, hepatitis B virus; HCV, hepatitis C virus; mALBI grade, modified ALBI grade; mBR, modified borderline resectable; NA, not available; NBNC, non‐HBV‐non‐HCV; RFA, radiofrequency ablation.

^a^ Median (interquartile range).

**FIGURE 5:**
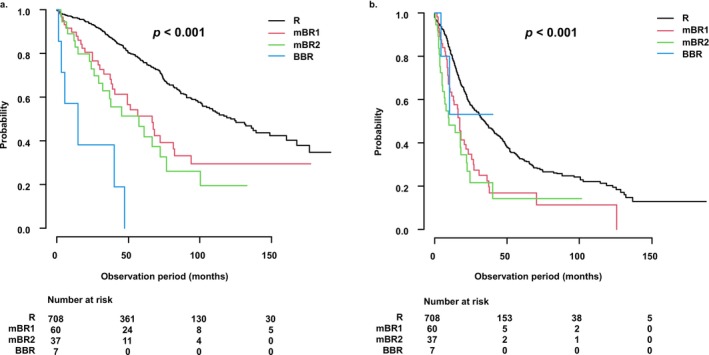
Survival and recurrence outcomes in HCC patients who underwent curative treatment according to the modified borderline resectable (mBR) criteria. (a) Among patients who underwent curative treatment (R: *N* = 708; mBR1: *N* = 60; mBR2: *N* = 37; BBR: *n* = 7), overall survival (OS) progressively declined with worsening mBR classification (median OS: R vs. mBR1 vs. mBR2 vs. BBR = 121.3 vs. 66.5 vs. 57.4 vs. 15.0 months; *p* < 0.001). (b) Similarly, recurrence‐free survival (RFS) also significantly decreased across the mBR subgroups (median RFS: R vs. mBR1 vs. mBR2 vs. BBR = 34.6 vs. 17.4 vs. 9.9 vs. not applicable [NA]; *p* < 0.001). BBR, boldly borderline resectable; Cur, curative treatment; HCC, hepatocellular carcinoma; mBR, modified borderline resectable; NA, not applicable; OS, overall survival; R, resectable; RFS, recurrence‐free survival.

The Kaplan–Meier curves demonstrated a clear stratification of OS in the Cur group among the four subgroups (R, mBR1, mBR2, and BBR), with progressively worse outcomes observed as the classification advanced from R to BBR (Figure 5). The median OS was significantly longer in the R group compared with the other groups (log‐rank test, *p* < 0.001).

Univariate Cox proportional hazards analysis revealed that older age, higher mALBI grade 2b, poor PS (≥ 2), elevated AFP (AFP ≥ 100 ng/mL), elevated DCP (DCP ≥ 100 mAU/mL), presence of PH, higher mBR classification, and NC were significantly associated with worse OS (all *p* < 0.001, Table 6). In the multivariate analysis, age (HR: 1.022, 95% CI: 1.010–1.035, *p* < 0.001), mALBI grade 2b (HR: 2.044, 95% CI: 1.558–2.682, *p* < 0.001), PS ≥ 2 (HR: 1.535, 95% CI: 1.045–2.253, *p* = 0.029), AFP ≥ 100 ng/mL (HR: 1.276, 95% CI: 1.005–1.620, *p* = 0.045), and DCP ≥ 100 mAU/mL (HR: 1.588, 95% CI: 1.254–2.011, *p* < 0.001) remained independent prognostic factors of OS. Regarding mBR classification, compared with the R group, patients classified as having mBR1, mBR2, and BBR showed significantly poorer OS with hazard ratios of 1.820 (95% CI: 1.226–2.703, *p* = 0.003), 2.459 (95% CI: 1.524–3.968, *p* < 0.001), and 9.009 (95% CI: 3.883–20.900, *p* < 0.001), respectively. Among the treatment modalities, NC (HR: 2.982, 95% CI: 1.759–5.057, *p* < 0.001) and BSC (HR: 2.851, 95% CI: 1.473–5.519, *p* = 0.002) were independently associated with poor OS compared with Cur. Notably, a significant interaction effect was observed for the combination of mBR1 and BSC (HR: 8.402, 95% CI: 2.356–29.970, *p* = 0.001), with an extremely poor prognosis observed for these patients (Table 6).

**TABLE 6:** Univariate and multivariable Cox proportional hazards analyses for overall survival.

Factors	Univariative analysis			Multivariable analysis		
	Hazard ratio	95% CI	*p*	Hazard ratio	95% CI	*p*
Age	1.030	1.019–1.042	< 0.001	1.022	1.010–1.035	< 0.001
Sex						
Female	0.921	0.734–1.156	0.477	0.822	0.647–1.043	0.107
Etiology						
Non‐Viral	1.201	0.974–1.480	0.086	1.000	0.795–1.257	0.998
mALBI grade						
2b	2.390	1.906–2.998	< 0.001	2.044	1.558–2.682	< 0.001
Performance status						
≥ 2	2.334	1.635–3.332	< 0.001	1.535	1.045–2.253	0.029
AFP						
≥ 100 ng/mL	1.696	1.358–2.117	< 0.001	1.276	1.005–1.620	0.045
DCP						
≥ 100 mAU/mL	2.366	1.932–2.898	< 0.001	1.588	1.254–2.011	< 0.001
PH	1.463	1.169–1.830	< 0.001	1.145	0.873–1.503	0.329
mBR classification (Ref = *R*)						
mBR1	2.550	1.882–3.455	< 0.001	1.820	1.226–2.703	0.003
mBR2	4.185	3.193–5.485	< 0.001	2.459	1.524–3.968	< 0.001
BBR	12.780	8.308–19.660	< 0.001	9.009	3.883–20.900	< 0.001
Treatment (Ref = C)						
NC	5.099	3.975–6.542	< 0.001	2.982	1.759–5.057	< 0.001
BSC	6.743	4.379–10.380	< 0.001	2.851	1.473–5.519	0.002
mBR classification × treatment (Ref R × C)						
mBR1 × NC	0.564	0.258–1.231	0.150	0.762	0.346–1.678	0.501
mBR2 × NC	0.456	0.220–0.943	0.034	0.575	0.276–1.198	0.139
BBR × NC	0.359	0.124–1.035	0.058	0.471	0.157–1.418	0.181
mBR1 × BSC	5.195	1.509–17.880	0.009	8.402	2.356–29.970	0.001
mBR2 × BSC	1.202	0.427–3.382	0.727	1.474	0.512–4.243	0.472
BBR × BSC	0.473	0.084–2.661	0.395	0.765	0.132–4.422	0.765

Abbreviations: AFP, alpha‐fetoprotein; AFP‐L3, ALBI score, albumin‐bilirubin score; BBR, boldly borderline resectable; BR, borderline resectable; BSC, best supportive care; Cur, curative therapy; DCP, des‐gamma‐carboxy prothrombin; mALBI grade, modified ALBI grade; mBR, modified borderline resectable; NC, non‐curative therapy; PH, portal hypertension; R, resectable; RFA, radiofrequency ablation.

Furthermore, in the subgroup analysis according to mBR classification, both NC and BSC were associated with significantly worse OS compared with Cur in the R group (NC: HR, 5.060; 95% CI, 3.033–8.442; *p* < 0.001; BSC: HR, 5.380; 95% CI, 2.803–10.320; *p* < 0.001). In the mBR1 group, NC and BSC were also significantly associated with poorer survival (NC: HR, 2.057; 95% CI, 1.103–3.836; *p* = 0.023; BSC: HR, 26.560; 95% CI, 7.323–96.360; *p* < 0.001). Similarly, in the mBR2 group, both NC and BSC showed significantly worse OS relative to Cur (NC: HR, 1.868; 95% CI, 1.097–3.180; *p* = 0.021; BSC: HR, 4.256; 95% CI, 1.850–9.795; *p* < 0.001). In contrast, in the BBR group, neither NC nor BSC demonstrated a statistically significant association with OS when compared with Cur (NC: HR, 1.300; 95% CI, 0.503–3.364; *p* = 0.588; BSC: HR, 1.783; 95% CI, 0.347–9.169; *p* = 0.489) (Table S5).

## 4 Discussion

This study demonstrated that the mBR criteria, which assign patients with EHM to an independent group, provide a clinically useful framework for prognostic stratification of naïve patients with HCC. OS progressively worsened with advancing mBR class, and this trend was also observed among surgically treated patients. Similarly, deteriorations in liver function, PS, and tumor marker profiles were observed with more advanced mBR classification. Curative treatment was associated with significantly better outcomes in the R, mBR1, and mBR2 groups, while patients in the BBR group had a uniformly poor prognosis regardless of treatment modality.

Consistent with previous reports [34] that used the original BR‐HCC criteria, the mBR criteria used in the present study demonstrated effective prognostic stratification. Additionally, patients with EHM had a significantly worse prognosis than those without EHM (Figure 1). These findings suggest that, besides selected cases with well‐controlled intrahepatic disease [11, 12] or limited pulmonary metastases [13] as previously reported, the therapeutic benefit—regardless of curative or non‐curative intent—was limited in patients with HCC and EHM at initial treatment. With recent advances in systemic therapy, including immunotherapy, the importance of conversion therapy as a treatment option for improving prognosis has increased in patients who have achieved tumor shrinkage or localization after systemic therapy [9, 35], even in those with advanced HCC accompanied by EHM. Further studies are warranted to establish effective treatment strategies for this subgroup including treatment‐naïve and previously‐treated with systemic therapy groups.

In the present study, patients classified into mBR1 and mBR2 groups—categories beyond SR indication in the BCLC staging system [18]—who received curative treatments, demonstrated the most favorable OS, although early recurrence was observed in the mBR1 and mBR2 groups. These findings suggest that curative interventions may offer clinical benefit even in selected patients within the advanced mBR groups. Notably, curative treatments were more frequently selected for patients with well‐preserved liver function and good PS, indicating that these baseline factors play a critical role in patient selection. In addition, rapid progression of systemic treatments including immune checkpoint inhibitors is considered to have contributed to the improved prognosis in such patients. Therefore, considering recent development of effective systemic therapies, it is worthwhile to actively consider curative treatment even in patients with advanced resectability classification, if they have sufficient liver function reserve and are in good general condition.

Our study interaction analysis revealed that the prognostic impact of mBR classification varied depending on the treatment modality. Notably, a significant interaction was observed between mBR1 and BSC, indicating that BSC was associated with a disproportionately poor prognosis among patients classified as having mBR1. Patients in the mBR1 group are generally considered to have intermediate disease status, where active treatments such as surgical resection or local ablation could potentially improve survival. Therefore, when such patients receive BSC instead of active interventions, their prognosis may deteriorate markedly due to the absence of tumor control. In contrast, patients in the BBR group have extremely advanced disease, and their prognosis remains poor regardless of the treatment modality, which may explain why no significant interaction was observed in this group. These findings highlight the importance of treatment selection based on mBR classification and suggest that curative or disease‐controlling interventions should be considered whenever possible for mBR1 patients to improve outcomes.

This study had some limitations. First, it was retrospective in nature and conducted at a single center, which may limit the generalizability of the findings. Second, treatment allocation was not randomized, and this may have introduced selection bias, particularly in assigning to Cur versus NC. Third, the number of patients in the BBR group was relatively small, which may have limited the statistical power to detect differences among treatment subgroups. Fourth, there was no evaluation of the prognosis in patients who underwent curative conversion therapy following systemic treatment. Finally, molecular or genetic data, which could further refine prognostic stratification, were not included in the present analysis.

Because the number of patients classified as having BBR was relatively small, the statistical power to detect significant differences may have been limited. Therefore, the non‐significant results observed in this group should be interpreted with caution, and further studies with larger sample sizes are needed to confirm these findings.

Although some patients classified into the resectable group in this study might also have been potential candidates for liver transplantation, this is strictly regulated under the Japanese national health insurance system and generally limited to patients meeting the Japanese treatment guidelines [31]. At our institution, liver transplantation is considered primarily for younger patients, and in practice, very few patients actively pursue transplantation as an initial treatment option. This should be recognized as a limitation of our study.

Because the prognosis in HCC patients with EHM was poor regardless of treatment modality. Therefore, in carefully selected patients with EHM, aggressive SR might be considered as a conversion treatment following systemic therapy, when feasible. Notably, the present mBR criteria demonstrated that aggressive treatment may improve the prognosis in patients without EHM.

In conclusion, despite potential selection bias, aggressive treatments may be a viable initial therapeutic option in selected patients with unresectable HCC, including those with BCLC stage B or C, classified as mBR1 or mBR2.

## Author Contributions

**Fujimasa Tada:** conceptualization, investigation, writing – original draft, methodology, validation, visualization, writing – review and editing, data curation, project administration, software, supervision, formal analysis, resources. **Atsushi Hiraoka:** conceptualization, investigation, methodology, validation, writing – review and editing, writing – original draft, data curation. **Hideko Ohama:** data curation. **Mai Saito:** data curation. **Yuka Kimura:** data curation. **Ayaka Nakamura:** data curation. **Toru Usui:** data curation. **Kanako Kato:** data curation. **Kei Onishi:** data curation. **Shogo Kitahata:** data curation. **Kozue Kanemitsu‐Okada:** data curation. **Tomoe Kawamura:** data curation. **Taira Kuroda:** data curation. **Naho Ishimura:** data curation. **Jun Hanaoka:** data curation. **Jota Watanabe:** data curation. **Hiromi Ohtani:** data curation. **Teruki Miyake:** data curation. **Osamu Yoshida:** data curation. **Masashi Hirooka:** data curation. **Hideki Miyata:** data curation. **Eiji Tsubouchi:** data curation. **Masanori Abe:** data curation. **Tomoyuki Ninomiya:** data curation. **Yoichi Hiasa:** data curation.

## Funding

The authors have nothing to report.

## Ethics Statement

The protocol for this research was approved by the Ethics Committee of Ehime Prefectural Central Hospital (Approval No. 26–11) and conducted in accordance with the principles of the Declaration of Helsinki. The study adhered to the institutional ethical guidelines, and the requirement for written informed consent was waived due to the retrospective nature of the research, use of anonymized data, and minimal risk to participants. Details of the ethical approval are available on the hospital's official website.

## Conflicts of Interest

Atsushi Hiraoka received lecture fees from Eli Lilly, AstraZeneca, and Chugai. The other authors declare no conflicts of interest associated with this study.

## Supporting information


**Table S1:** Original and modified oncological criteria for resectability of hepatocellular carcinoma in patients in Japan presented in 2023 expert consensus statement.

**Table S2:** Pairwise comparisons among four groups with Holm‐adjusted *p*‐values.

**Table S3:** Baseline clinical characteristics of patients in the modified borderline resectable type1 (mBR1) group stratified by treatment strategy based on mBR criteria.

**Table S4:** Baseline clinical characteristics of patients in the modified borderline resectable type 2 (mBR2) group stratified by treatment strategy based on mBR criteria.

**Table S5:** Univariate Cox proportional hazards analyses for overall survival.

## Data Availability

All data associated with the present study will be available from the corresponding author upon reasonable request.
