# Supplementary material for: Prognostic Stratification of Initial Treatments for Hepatocellular Carcinoma Using a Modified Borderline Resectable Classification
Source: Cancer Med. 2025 Dec 17;14(24):e71470. doi: 10.1002/cam4.71470 (PMC12710435; doi:10.1002/cam4.71470)
Supplement: Supplementary file 1 — Table S1: Original and modified oncological criteria for resectability of hepatocellular carcinoma in patients in Japan presented in 2023 expert consensus statement. [file CAM4-14-e71470-s004.docx]

# Supplementary Table 1. Original and modified oncological criteria for resectability of hepatocellular carcinoma in patients in Japan presented in 2023 expert consensus statement

| Original oncological criteria for resectability of hepatocellular carcinoma (Original borderline resectable [BR] criteria) | | |
| --- | --- | --- |
| Resectable (R) | Borderline resectable 1 (BR1) | Borderline resectable 2 (BR2) |
| Solitary lesion  (no size limit) | x | x |
| Multiple lesions:  2-3 nodules, each ≤3 cm | Multiple lesions exceeding criteria of R, but ≤5 nodules or ≤5 cm | Multiple lesions,  > 5 nodules or >5 cm |
| Vp0-1/Vv0-1/B0-1 | Vp2-3/Vv2/B2-3 | Vp4/Vv3/B4 |
| x | Localized extrahepatic spreading:  solitary lymph node at no. 3, 8, or 12,  localized peritoneal dissemination,  unilateral adrenal metastasis,  oligometastatic lung tumor | Extrahepatic spreading |

| Modified oncological criteria for resectability of hepatocellular carcinoma (Modified borderline resectable [BR] criteria) | | | |
| --- | --- | --- | --- |
| Resectable (R) | Modified borderline resectable 1 (mBR1) | Modified borderline resectable 2 (mBR2) | Boldly borderline resectable (BBR) |
| Solitary lesion (no size limit) | x | x | Any |
| Multiple lesions: 2-3 nodules, each ≤3 cm | Multiple lesions exceeding criteria of R, but ≤5 nodules or ≤5 cm | Multiple lesions,  > 5 nodules or >5 cm | Any |
| Vp 0-1/Vv 0-1/B 0-1 | Vp 2-3/Vv 2/B 2-3 | Vp 4/Vv 3/B 4 | Any |
| x | x | x | Extrahepatic spreading  (including localized extrahepatic spreading) |

BBR, boldly borderline resectable; mBR, modified borderline resectable; R, resectable
