# Supplementary material for: Prognostic Stratification of Initial Treatments for Hepatocellular Carcinoma Using a Modified Borderline Resectable Classification
Source: Cancer Med. 2025 Dec 17;14(24):e71470. doi: 10.1002/cam4.71470 (PMC12710435; doi:10.1002/cam4.71470)
Supplement: Supplementary file 2 — Table S2: Pairwise comparisons among four groups with Holm‐adjusted p‐values. [file CAM4-14-e71470-s002.docx]

# Supplementary Table 2. Pairwise comparisons among four groups with Holm-adjusted *p*-values

| Comparison | Holm-adjusted *p*-value | Statistical significance (α = 0.05) |
| --- | --- | --- |
| Group R vs Group mBR1 | 5.4 × 10⁻¹⁰ | Significant |
| Group R vs Group mBR2 | < 2.0 × 10⁻¹⁶ | Significant |
| Group R vs Group BBR | < 2.0 × 10⁻¹⁶ | Significant |
| Group mBR1 vs Group mBR2 | 0.0252 | Significant |
| Group mBR1 vs Group BBR | 7.0 × 10⁻⁹ | Significant |
| Group mBR2 vs Group BBR | 0.00019 | Significant |

BBR, boldly borderline resectable; mBR, modified borderline resectable; R, resectable.
