# Supplementary material for: Prognostic Stratification of Initial Treatments for Hepatocellular Carcinoma Using a Modified Borderline Resectable Classification
Source: Cancer Med. 2025 Dec 17;14(24):e71470. doi: 10.1002/cam4.71470 (PMC12710435; doi:10.1002/cam4.71470)
Supplement: Supplementary file 3 — Table S3: Baseline clinical characteristics of patients in the modified borderline resectable type1 (mBR1) group stratified by treatment strategy based on mBR criteria. [file CAM4-14-e71470-s005.docx]

# Supplementary Table 3. Baseline clinical characteristics of patients in the modified borderline resectable type1 (mBR1) group stratified by treatment strategy based on mBR criteria

|  | curative (n=60) | non-curative (n=25) | BSC (n=10) | p value |
| --- | --- | --- | --- | --- |
| Age, years* | 72 (64 - 79) | 73 (70 - 80) | 77 (76 – 85) | 0.182 |
| Sex, male: female | 43: 17 | 18: 7 | 5: 5 | 0.368 |
| Etiology, HCV: HBV: HBV+HCV: Alcohol: NBNC | 35: 10: 1: 6: 8 | 12: 1: 0: 4: 8 | 5: 0: 0: 2: 3 | 0.315 |
| BMI, kg/m^2^* | 22.9 (21.3 – 24.7) | 23.5 (21.7 – 26.5) | 21.5 (19.8 – 22.7) | 0.133 |
| ECOG PS, 0: 1: 2: 3: 4 | 46: 13: 0: 1: 0 | 16: 5: 3:1: 0 | 4: 3: 1: 1: 1 | 0.012 |
| PS 0/1: 2/3/4, (%) | 59: 1 (98.3: 1.7) | 21: 4 (84.0: 16.0) | 7: 3 (70.0: 30.0) | 0.003 |
| ALBI score* | -2.64 (-2.90 to -2.48) | -2.44 (-2.68 to -2.06) | -2.37 (-2.52 to -2.29) | <0.001 |
| mALBI grade 1:2a:2b:3 | 31: 23: 6: 0 | 7: 7: 11: 0 | 0: 8: 2: 0 | <0.001 |
| AST, U/L* | 47 (29 - 75) | 44 (35 – 62) | 65 (46 – 113) | 0.311 |
| ALT, U/L* | 45 (22 – 63) | 36 (24 – 56) | 54 (45 – 100) | 0.189 |
| Platelets, 10^4^/µL* | 13.1 (9.8 – 16.7) | 13.5 (9.3 – 17.2) | 15.1 (11.4 – 17.9) | 0.619 |
| Total bilirubin, mg/dL* | 0.6 (0.5 – 0.9) | 1.2 (0.7 – 1.5) | 1.0 (0.8 – 1.4) | <0.001 |
| Albumin, g/dL* | 4.0 (3.7 - 4.2) | 3.7 (3.4 – 3.9) | 3.8 (3.6 – 3.9) | 0.013 |
| Prothrombin time, %* | 89.5 (84.5 – 96.5) | 83.0 (77.9 – 89.0) | 83.5 (79.6 – 93.5) | 0.053 |
| Tumor size (maximum), cm* | 4.0 (3.3 – 4.5) | 2.8 (2.0 – 4.3) | 4.5 (3.9 – 9.3) | 0.001 |
| Tumor diameter ≥ 2cm, n (%) | 57 (95.0) | 20 (80.0) | 10 (100) | 0.003 |
| Tumor number, single: multiple | 8: 52 | 1: 24 | 4: 6 | 0.020 |
| Portal invasion (vp0/vp1/vp2/vp3/vp4), n (%) | 49/1/8/2/0  (81.7/1.7/13.3/3.3/0) | 19/1/4/1/0  (76.0/4.0/16.0/4.0/0) | 7/0/0/3/0  (70.0/0/0/30.0/0) | 0.055 |
| Venous invasion (vv0/vv1/vv2/vv3), n (%) | 59/1/0/0 (98.3/1.7/0/0) | 23/0/2/0 (92.0/0/8.0/0) | 9/0/1/0 (90.0/0/10.0/0) | 0.204 |
| Biliary invasion (b0/b1/b2/b3/b4), n (%) | 60/0/0/0/0 (100/0/0/0/0) | 25/0/0/0/0 (100/0/0/0/0) | 10/0/0/0/0 (100/0/0/0/0) | NA |
| AFP, ng/mL* | 22.4 (7.0 – 169.5) | 11.3 (4.6 – 116.3) | 510.9 (63.3 – 1531.6) | 0.011 |
| AFP ≥ 100ng/mL, n (%) | 19 (31.7) | 8 (32.0) | 7 (70.0) | 0.058 |
| AFP-L3, %* | 7.4 (1.5 – 30.0) | 3.9 (0.5 – 12.4) | 6.4 (3.0 – 9.3) | 0.001 |
| AFP-L3 ≥ 10%, n (%) | 26 (44.1) | 9 (36.0) | 3 (33.3) | 0.702 |
| DCP, mAU/mL* | 506 (75 – 1845) | 105 (48 – 690) | 4794 (1502 – 20644) | 0.005 |
| DCP ≥ 100mAU/mL, n (%) | 42 (70.0) | 13 (52.0) | 9 (90.0) | 0.074 |

*Median (interquartile range). AFP, alpha-fetoprotein; AFP-L3, lens culinaris agglutinin-reactive AFP; ALBI score, albumin-bilirubin score; ALT, alanine aminotransferase; AST, aspartate aminotransferase; BMI, body mass index; BR, borderline resectable; BSC, best supportive care; DCP, des-gamma-carboxy prothrombin; ECOG PS, Eastern Cooperative Oncology Group performance status; HBV, hepatitis B virus; HCV, hepatitis C virus; mALBI grade, modified ALBI grade; mBR, modified borderline resectable; NA, not available; NBNC, non-HBV-non-HCV; RFA, radiofrequency ablation.
