# Supplementary material for: Prognostic Stratification of Initial Treatments for Hepatocellular Carcinoma Using a Modified Borderline Resectable Classification
Source: Cancer Med. 2025 Dec 17;14(24):e71470. doi: 10.1002/cam4.71470 (PMC12710435; doi:10.1002/cam4.71470)
Supplement: Supplementary file 4 — Table S4: Baseline clinical characteristics of patients in the modified borderline resectable type 2 (mBR2) group stratified by treatment strategy based on mBR criteria. [file CAM4-14-e71470-s003.docx]

# Supplementary Table 4. Baseline clinical characteristics of patients in the modified borderline resectable type 2 (mBR2) group stratified by treatment strategy based on mBR criteria

|  | curative (n=37) | non-curative (n=68) | BSC (n=24) | p value |
| --- | --- | --- | --- | --- |
| Age, years* | 67 (62 - 75) | 74 (67 - 79) | 80 (71 – 83) | 0.001 |
| Sex, male: female | 30: 7 | 51: 17 | 18: 6 | 0.761 |
| Etiology, HCV: HBV: HBV+HCV: Alcohol: NBNC | 14: 1: 0: 8: 14 | 27: 6: 1: 15: 19 | 11: 2: 0: 1: 10 | 0.523 |
| BMI, kg/m^2^* | 24.1 (22.1 – 26.7) | 23.8 (22.5 – 26.2) | 23.2 (21.3 – 26.5) | 0.824 |
| ECOG PS, 0: 1: 2: 3: 4 | 33: 4: 0: 0: 0 | 51: 7: 8:2: 0 | 10: 4: 6: 3: 1 | 0.002 |
| PS 0/1: 2/3/4, (%) | 37: 0 (100: 0) | 58: 10 (85.3: 14.7) | 14: 10 (58.3: 41.7) | <0.001 |
| ALBI score* | -2.61 (-2.74 to -2.43) | -2.34 (-2.60 to -2.09) | -2.29 (-2.39 to -2.02) | <0.001 |
| mALBI grade 1:2a:2b:3 | 21: 10: 6: 0 | 16: 21: 31: 0 | 3: 10: 11: 0 | 0.001 |
| AST, U/L* | 55 (38 - 78) | 60 (41 – 86) | 72 (49 – 117) | 0.089 |
| ALT, U/L* | 43 (27 – 64) | 43 (27 – 69) | 38 (29 – 58) | 0.884 |
| Platelets, 10^4^/µL* | 18.9 (14.9 – 24.2) | 13.2 (8.5 – 18.5) | 17.3 (14.2 – 24.8) | <0.001 |
| Total bilirubin, mg/dL* | 0.7 (0.6 – 0.9) | 0.9 (0.6 – 1.3) | 0.8 (0.7 – 1.1) | 0.099 |
| Albumin, g/dL* | 3.9 (3.7 - 4.1) | 3.7 (3.4 – 4.0) | 3.6 (3.3 – 3.7) | 0.001 |
| Prothrombin time, %* | 88.0 (80.0 – 95.4) | 89.0 (78.8 – 95.4) | 89.0 (82.3 – 97.0) | 0.89 |
| Tumor size (maximum), cm* | 8.0 (6.0 – 10.0) | 4.9 (3.0 – 7.6) | 8.3 (5.2 – 11.3) | <0.001 |
| Tumor diameter ≥ 2cm, n (%) | 36 (97.3) | 61 (89.7) | 24 (100) | 0.115 |
| Tumor number, single: multiple | 1: 36 | 2: 66 | 4: 20 | 0.026 |
| Portal invasion (vp0/vp1/vp2/vp3/vp4), n (%) | 30/1/4/1/1  (81.1/2.7/10.8/2.7/2.7) | 50/0/4/7/7  (73.5/0/5.9/10.3/10.3) | 13/0/2/2/7  (54.2/0/8.3/8.3/29.2) | 0.05 |
| Venous invasion (vv0/vv1/vv2/vv3), n (%) | 36/0/1/0 (97.3/0/2.7/0) | 66/0/2/0 (97.1/0/2.9/0) | 21/0/1/2 (87.5/0/4.2/8.3) | 0.06 |
| Biliary invasion (b0/b1/b2/b3/b4), n (%) | 37/0/0/0/0 (100/0/0/0/0) | 66/1/0/1/0 (97.1/1.5/0/1.5/0) | 23/0/1/0/0 (95.8/0/4.2/0/0) | 0.401 |
| AFP, ng/mL* (95.8/0/4.2/0/0 | 60.6 (11.1 – 2140.6) | 56.9 (10.0 – 619.2) | 1341.8 (32.0 – 7183.8) | 0.109 |
| AFP ≥ 100ng/mL, n (%) | 16 (43.2) | 29 (42.6) | 15 (62.5) | 0.219 |
| AFP-L3, %* | 25.1 (6.5 – 54.6) | 10.2 (2.2 – 42.8) | 32.3 (15.2 – 74.8) | 0.021 |
| AFP-L3 ≥ 10%, n (%) | 21 (56.8) | 33 (48.5) | 16 (69.6) | 0.206 |
| DCP, mAU/mL* | 7141 (768 – 22972) | 870 (72 – 5705) | 6062 (1912 – 44414) | 0.001 |
| DCP ≥ 100mAU/mL, n (%) | 35 (94.6) | 49 (72.1) | 21 (87.5) | 0.013 |

*Median (interquartile range). AFP, alpha-fetoprotein; AFP-L3, lens culinaris agglutinin-reactive AFP; ALBI score, albumin-bilirubin score; ALT, alanine aminotransferase; AST, aspartate aminotransferase; BMI, body mass index; BR, borderline resectable; BSC, best supportive care; DCP, des-gamma-carboxy prothrombin; ECOG PS, Eastern Cooperative Oncology Group performance status; HBV, hepatitis B virus; HCV, hepatitis C virus; mALBI grade, modified ALBI grade; mBR, modified borderline resectable; NBNC, non-HBV-non-HCV; RFA, radiofrequency ablation.
