# Supplementary material for: Prognostic Stratification of Initial Treatments for Hepatocellular Carcinoma Using a Modified Borderline Resectable Classification
Source: Cancer Med. 2025 Dec 17;14(24):e71470. doi: 10.1002/cam4.71470 (PMC12710435; doi:10.1002/cam4.71470)
Supplement: Supplementary file 5 — Table S5: Univariate Cox proportional hazards analyses for overall survival. [file CAM4-14-e71470-s001.docx]

Supplementary Table 5. Univariate Cox Proportional Hazards Analyses for Overall Survival

| Factors | Treatment (Ref=Cur) | Hazard ratio | 95% CI | p-value |
| --- | --- | --- | --- | --- |
| R (n=789) | NC | 5.060 | 3.033 – 8.442 | <0.001 |
|  | BSC | 5.380 | 2.803 – 10.320 | <0.001 |
| mBR1 (n=95) | NC | 2.057 | 1.103 – 3.836 | 0.023 |
|  | BSC | 26.560 | 7.323 – 96.360 | <0.001 |
| mBR2 (n=128) | NC | 1.868 | 1.097 – 3.180 | 0.021 |
|  | BSC | 4.256 | 1.850 – 9.795 | <0.001 |
| BBR (n=44) | NC | 1.300 | 0.503 – 3.364 | 0.588 |
|  | BSC | 1.783 | 0.347 – 9.169 | 0.489 |

BBR, boldly borderline resectable; BR, borderline resectable; BSC, best supportive care; Cur, curative therapy; mBR, modified borderline resectable; NC, non-curative therapy; R, resectable; Ref, reference.
